# Account of the Effects of a Large Dose of Emetic Tartar; with Remarks

**Published:** 1788

**Authors:** William Blackburne

**Affiliations:** Member of the Royal College of Physicians, London


### VII. *Account of the Effects of a large Dose of Emetic Tartar; with Remarks. Communicated in a Letter to* Samuel Foart Simmons, *M. D. F. R. S. by* William Blackburne, *M. D. F. A. S. Member of the Royal College of Physicians, London.*

A Young lady, nineteen years of age, of a very delicate frame, and of a fair florid complexion, was feized on the 24th of June, 1787, with fhivering, followed by heat, thirft, head ach, and pain in all her limbs. Thefe fymptoms had been preceded by an hæmorrhage from the uterus, which, after fubfifting for fome weeks, had declined about a fortnight before the above-mentioned attack of fever, and had left her in a ftate of great debility.

On the evening of the 24th, her friends had given to her fifteen grains of tartar emetic, inftead of the fame quantity of the powder of ipecacuanha. The next morning my attendance was requefted, and I found her labouring under the following fymptoms.—Her countenance was extremely pale; and as well as the reft of her body, was bedewed with a cold clammy fweat. Convulfive twitchings affected fucceffively almoft every mufcle of her face.

Catchings

Catchings, and fubfultus tendinum, were equally obvious and frequent in her hands and arms. Her pulfe was quick, weak, and tremulous; her tongue moift and clean. The antimony had induced moft violent ficknefs, and inceffant vomiting, during a great part of the preceding night, and fhe had voided four liquid ftools in the courfe of the laft twelve hours. Her breathing was extremely difficult, and being raifed up on pillows to facilitate refpiration, whenever fhe attempted to move her head, or bend it forwards, the motion of it exactly refembled a paralytic tremor. The fame fort of tremor accompanied every effort fhe made to lift her hands; and occafional faintings were added to this train of formidable appearances.

As the caufe of thefe fymptoms was fufficiently ftriking, and the track I had to purfue feemed to be no lefs plain, I immediately ordered a cordial compofed of Mofch, Sal c. cervi, Paregoric elixir, and Mint water in due proportions, to be exhibited at fuch intervals as the patient's ftomach could bear. In the evening I found her confiderably relieved. She had taken fome little nourifhment in the way of broth, as had been directed; the colour had in fome degree degree returned in her cheeks; a kindly warmth diffufed itfelf over her whole body, and fhe breathed with lefs difficulty: but her pulfe was ftill quick and weak, though lefs tremulous, and the convulfive motions and fubfultus tendinum were not diminifhed.

As the medicine had agreed with her perfectly well, it was ordered to be continued, and, at the fame time, I recommended an enema to be adminiftered, compofed of broth, with the addition of half an ounce of caftor oil, and thirty drops of laudanum. The next morning fhe appeared to be refrefhed, having had fome hours fleep; all the convulfive fymptoms were now much abated, but the tremor of her head and hands was ftill confiderable.

As the ufe of the bark feemed now to be indicated, it was given in the draught inftead of the mint water. By continuing this cordial and ftrengthening medicine for fome time, (having alfo recourfe to the occafional aid of Madeira) and by interpofing two gentle dofes of the caftor oil, the patient gradually recovered her ftrength, and regained her ufual ftate of health.

It muft not pafs unobferved, that the tremor of the head was the laft remaining fymptom; tom; and that in her convalefcent ftate this delicate fufferer languifhed both a longer time, and under a greater degree of feeblenefs, after this fhock of the nervous fyftem, than I have ever known to have happened in perfons recovering from fevers, even of long duration and of the loweft kind.

It may not be unfeafonable to offer one or two remarks on the cafe above recited.

Firft, It feems reafonable to conclude, that the tartar emetic, in this inftance, operated as a poifon; and although the efforts to expel it were violent, yet a fufficient portion of it remained in the ftomach and bowels to interrupt the action of the moving fibres, and to affect the nervous fyftem, in a manner truly alarming. On the fubject of poifons, it is not very foreign to our prefent difquifition to obferve, that certain mineral poifons, when applied to the human body, exert a mode of action peculiar to each, and differing from others. Thus, lead induces a particular kind of colic followed by palfy; arfenic acts as a cauftic very foon after its reception into the ftomach, and brings on, with exquifite pain, ulceration and mortification; copper occafions violent ficknefs and vomiting, with pain and inflammation of that organ; mercury mercury, in general, as well as lead, copper, and antimony, requires to be in combination with an acid folvent, before it acts fpecifically as a poifon, and then its effects (as in the form of corrofive fublimate) are fimilar to thofe of copper: I have, however, known inftances where it has been introduced into the fyftem too abundantly in its crude divided ftate, and has given rife to moft obftinate palfies. But the operation of antimony, in cafes where it may be confidered as poifonous, is different from that of all the other mineral poifons I have mentioned; for it acts without pain, and after raifing certain efforts for its own expulfion, it prevents the complete effects of thefe by deftroying the nervous energy, fufpending the action of the moving fibres, and even taking life * away infidioufly, and as it were by ftealth.

Permit

* I can charge my recollection with two cafes in which the taking of tartar emetic proved fatal. The firft was that of a child who had fome trifling complaint in its bowels, attended with a purging, and apparently very flight gripes. Two grains of tartar emetic were given to this child as an emetic; but no fuch effect was produced. Cold fweats, infenfibility, tremors, and convulfions fhortly fucceeded the exhibition of this medicine, and notwithftanding the moft fpeedy efforts of art, the child died in a few hours. The fecond

 inftance

Permit me to offer one more obfervation with regard to poifons. Important diftinctions feem to be eftablifhed between vegetable and mineral poifons in their mode of affecting the human frame; and one difference confifts in this, that the latter do not generally affect the intellects, while thefe are almoft conftantly difturbed by the former. The deleterious effects of night-fhade, henbane, opium, &c. are fufficiently well known, and feem to confirm the obfervation juft now offered. If this is a fact generally to be depended on, though perhaps with fome exceptions, the farther inveftigation of it appears interefting, not only as a fubject of curious fpeculation, but as leading to a diftinct arrangement of the fpecific operation inftance was that of an apparently ftrong healthy woman, a farmer's wife, between fifty and fixty years of age, who in the year 1781 was feized with the Influenza. The fymptoms refembled thofe of pleurify, but were deemed fo mild as not to require bleeding. In this cafe a blifter was applied to the fide, and at night four grains of tartar emetic were given to the patient. Violent vomiting and purging enfued. On the fucceeding morning when I faw her, faintings in quick fucceffion, cold fweats, a finking and fcarce perceptible pulfe, with liquid ftools paffed infenfibly, were the urgent and alarming fymptoms that prevailed.

With operation of the various poifons (whether acting upon the moving fibres or the fluids) and from thence may bring us nearer to the difcovery of a certain and peculiar antidote to each.

*Spring Gardens,*
March 6, 1788.

---

### VIII. *Some Account of Tipioca; and of the Species of Ipecacuanha found near Rio Janeiro.*

WE have written the firft of thefe names as we find it written by Pifo, who in his work *de Indiæ utriufque re naturali et medica**, has given a particular account of the fubftance which, within thefe few years only, has been imported into this country under the name of *Tapioca.*

With difficulty fmall quantities of warm wine, brandy, &c., were fwallowed, by which fhe was a little revived; but fhe funk at laft under the baneful effects of the tartar emetic, and died the next evening. She had fuffered no previous evacuations, and it was only the third day of her difeafe.

In the *Journal de Medecine* for October, 1787, we find an account of violent effects of a large dofe of emetic tartar, communicated by M. Gaterau, phyfician at Montauban; but he has not told us precifely what the dofe was.

